# Impact of the used solvent on the reconstitution efficiency of evaporated biosamples for untargeted metabolomics studies

**DOI:** 10.1007/s11306-019-1631-1

**Published:** 2020-03-02

**Authors:** Sascha K. Manier, Markus R. Meyer

**Affiliations:** Department of Experimental and Clinical Toxicology, Center for Molecular Signaling (PZMS), Institute of Experimental and Clinical Pharmacology and Toxicology, Saarland University, 66421 Homburg, Germany

**Keywords:** Untargeted metabolomics, Reconstitution, Method validation, Recovery

## Abstract

**Introduction:**

Untargeted metabolomics intends to objectively analyze a wide variety of compounds. Their diverse physicochemical properties make it difficult to choose an appropriate reconstitution solvent after sample evaporation without influencing the chromatography or hamper column sorbent integrity.

**Objectives:**

The study aimed to identify the most appropriate reconstitution solvent for blood plasma samples in terms of feature recovery, four endogenous compounds, and one selected internal standard.

**Methods:**

We investigated several reconstitution solvent mixtures containing acetonitrile and methanol to resolve human plasma extract and evaluated them concerning the peak areas of tryptophan-d_5_, glucose, creatinine, palmitic acid, and the phophatidylcholine PC(P-16:0/P-16:0), as well as the total feature count

**Results:**

Results indicated that acetonitrile containing 30% methanol was best suited to match all tested criteria at least for human blood plasma samples.

**Conclusion:**

Despite identifying the mixture of acetonitrile and methanol being suitable as solvent for human blood plasma extracts, we recommend to systematically test for an appropriate reconstitution solvent for each analyzed biomatrix.

**Electronic supplementary material:**

The online version of this article (10.1007/s11306-019-1631-1) contains supplementary material, which is available to authorized users.

## Introduction

Untargeted metabolomics (UT) is a technique that aims to objectively analyze biosamples without any limitation to their composition (Barnes et al. 2016). This intention leads to several challenges concerning chromatographical separation of the analytes as well as their detectability. Typically, samples are separated into their constituent compounds using normal phase and reversed phase chromatography to give consideration to the diverse physicochemical properties that they inherit. There are publications that aim to standardize the development of untargeted metabolomics methods as well as the reporting of the parameters in publications (Dudzik et al. 2018; Sumner et al. 2007). Concerning the laboratory workflow, these guideline include a comprehensive set of parameters from sample collection to metabolite extraction and matrix effects during ionization. One aspect that is usually not mentioned is the impact of the reconstitution solvent that is used to resolve the extract after its evaporation to dryness and which is usually the last step before injection onto the column of the chromatographic device. Lindahl et al. (2017) investigated the impact of several reconstitution solvents after extracting human plasma samples for separation using a reversed phase column. They came to the conclusion that the usage of pure water is most appropriate to resolve human plasma samples in their workflow to obtain an increased number of detected features as well as significant features. However as described above, typically untargeted metabolomics workflows use several column with physicochemical properties that are contrary to each other. The injection of pure water can lead to damages on normal phase columns such as those used for hydrophilic hydrophilic interaction liquid chromatography (HILIC). Acetonitrile is the only solvent that is recommended as a part of the mobile phase by the manufacturers for each of the used columns in this study and was therefore the first choice as a reconstitution solvent. The aim of this study was therefore to systematically test for a reconstitution solvent mixture that is most appropriate for reversed phase chromatography as well as normal phase chromatography.

## Experimental

### Chemicals and reagents

Pooled plasma samples (stabilized by citrate) were obtained from a regional blood bank. Methanol, ethanol, and acetonitrile (all LC–MS grade) were obtained from VWR (Darmstadt, Germany), ammonium formate, ammonium acetate, and formic acid from Merck (Darmstadt, Germany). l-Tryptophan-d_5_ was obtained from Alsachim (Illkirch-Graffenstaden, France). Water was purified with a Millipore filtration unit (18.2 Ω × cm water resistance).

### LC-HRMS/MS apparatus

Analysis was performed according to previously published studies (Helfer et al. 2015; Manier et al. 2018, 2019; Wagmann et al. 2017). Details can be found in the supplementary data.

### Sample preparation

For each mixture of reconstitution solvents, 100 µL of pooled human plasma samples were transferred into a reaction tube and precipitated using 400 µL of a mixture of methanol and ethanol (1:1, v/v) as recommended elsewhere (Bruce et al. 2009). The mixture contained 50 µmol tryptophan-d_5_ as internal standard. These samples were shaken for 2 min at 2000 rpm and subsequently centrifuged for 30 min at 15,000 rpm and 2 °C. 400 µL of the supernatant were transferred into a new reaction tube and evaporated to dryness using a vacuum centrifuge at 1400 rpm and room temperature. The obtained residues were reconstituted in different mixtures of acetonitrile and methanol containing 0, 10, 20, 30, 40, or 50% methanol. Each sample was prepared in triplicates. Quality Control (QC) samples that represent a common standard reconstitution solvent for each chromatographic condition used, were obtained by reconstituting the residue in eluent A and B (1:1, v/v) for analyses using a phenylhexyl column or eluent C and D (1:1, v/v) for analyses using HILIC. Five of these QC samples were prepared of each analysis. At last, a 1-µL aliquot was injected into the LC-HRMS/MS system.

### Data processing

Thermo Fisher LC-HRMS/MS RAW files were converted into mzXML files using Proteo Wizard (Adusumilli and Mallick 2017). Peak picking was performed using XCMS in an R environment (Smith et al. 2006; R Core Team 2019). Optimization of XCMS parameters was in accordance to a previously optimized strategy (Manier et al. 2018). Peak picking and alignment parameters are summarized in Table S1 in the supplementary data. After peak picking, tryptophan-d_5_ areas in full scan were evaluated for each sample group prepared with a different reconstitution mixture. To include a wider range of substance classes we additionally evaluated peak areas of creatinine and the phosphatidylcholine PC(P-16:0/P-16:0) for analyses using positive ionization, as well as glucose and palmitic acid for those using negative ionization mode. Total feature count was used to evaluate the number of features that were able to be detected by the analysis. For this parameter the number of features which peak area was not declared as not available (“NA”) was summed up for each analysis. Statistical evaluation was carried out using one-way ANOVA for variances as well as Welch’s two sample t-test for significance concerning group QC.

## Results and discussion

**Fig. 1:**
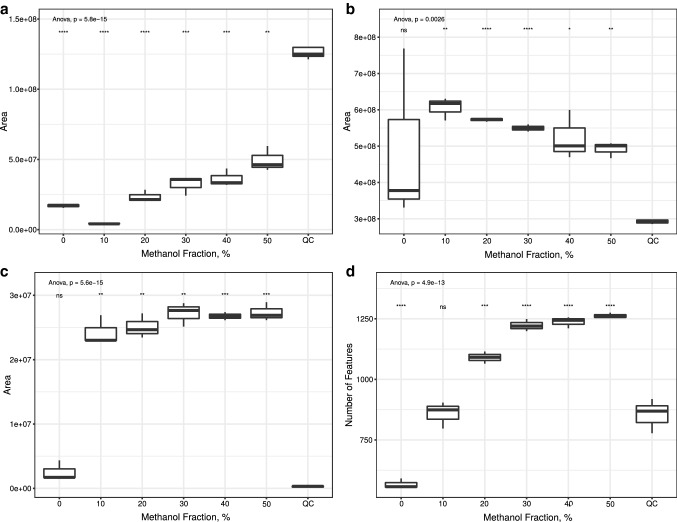
Results of analysis using phenylhexyl column in positive mode. Statistical evaluation was performed using one-way ANOVA and Welch’s two sample t-test comparing each group to QC. **a** Tryptophan-d_5_. **b** Creatinine. **c** PC(P-16:0/P-16:0). **d** Total feature count. *ns* not significant; *p < 0.05; **p < 0.01; ***p < 0.001; ****p < 0.0001

**Fig. 2:**
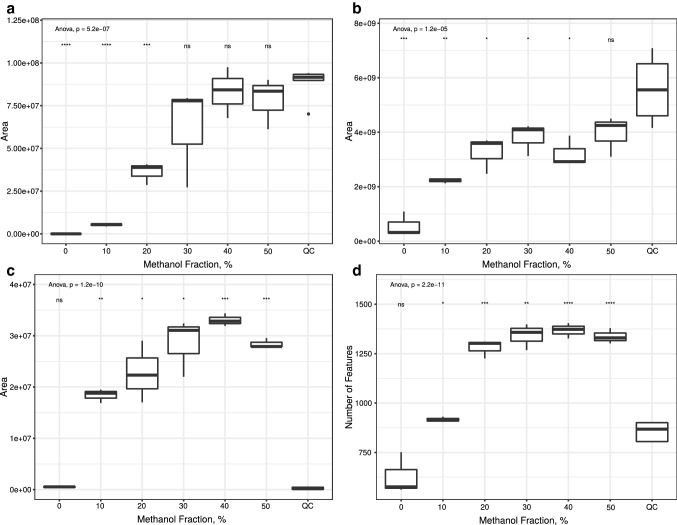
Results of analysis using HILIC in positive mode. Statistical evaluation was performed using one-way ANOVA and Welch’s two sample t-test comparing each group to QC. **a** Tryptophan-d_5_. **b** Creatinine. **c** PC(P-16:0/P-16:0). **d** Total feature count. *ns *not significant; *p < 0.05; **p < 0.01; ***p < 0.001; ****p < 0.0001

Results of analyses using positive ionization are displayed in Figs. 1 and 2. Those of analyses using negative ionization are displayed in Figs. S1 and S2 in the supplementary data. In every analysis the peak areas of the investigated compounds indicated a tendency to increase with an increasing amount of methanol that was used. While they were usually only detectible to a very low extend using 0, 10, or 20% methanol their area clearly increased using more than 20% methanol in the reconstitution solvent. This effect diminished for most compounds after the use of 30% methanol. One exception is the peak area of creatinine after using a phenylhexyl column and positive ionization (Fig. 1a). Its area first increases after using 20% methanol and subsequently decreases with a higher fraction of methanol in the reconstitution solvent. This effect is very likely the result of the low retention time of creatinine on reversed phase columns. Since most hydrophilic compounds eluate very early on this type of column there is an increased risk of ion suppression. It is notable that in the analysis using a phenylhexyl column and positive ionization mode (Fig. 1a), the area of tryptophan-d_5_ decreases at first after using 10% methanol before it increases further. This effect is also notable for glucose using HILIC in negative mode (Fig. S2a). This might be explained with the reconstitution solvent not only resolving the internal standard, but also other compounds that suppress the signal of tryptophan-d_5_ and glucose during ionization. Since solubility varies from compound to compound, the increasing fraction of methanol in the reconstitution solvent might resolve more tryptophan-d_5_ and glucose than the interfering analyte.

Concerning the total feature counts of these analyses, they also increase with higher amounts of methanol used for reconstitution. Again, this effect dimished after the usage of 30% methanol. These results underline the effect of methanol being beneficial for the reconstitution of analytes as seen above for those investigated in this study but indicate that this effect might not be limited to those analytes whose peak area has been monitored during this study but for several other analytes as well. This effect is most likely the reason of the high polarity of acetonitrile that leads to it not being able to spread on the polypropylene surface of the reaction tubes. Methanol has a considerably lower electric dipole moment and might act as a spreading mediator. Additional effects such as solubility of analytes in the reconstitution solvent are very likely relevant as well.

The total feature count was recently described as an inappropriate parameter to compare different methods in untargeted metabolomics (Mahieu et al. 2014), since features are not only the result of detected metabolites, but also of their isotopes, adducts and artifacts. Since in this study we used a highly standardized procedure and applied almost the same conditions on each sample the variability of the total feature count that is the result of different mechanisms is rather small compared to the variability that is introduced by the different concentrations of the metabolites. Given these conditions, such an increase in total feature counts can only hardly be explained by additional mechanisms of artifact formation that are introduced by adding methanol to a reconstitution solvent. In this study the increase of the total feature count is very likely the result of additional metabolites, that can be detected by adding methanol to the reconstitution solvent.

All of the above described results lead to the conclusion that different reconstitution mixtures have a huge effect on the recovery of analytes. Concerning the area of the investigated analytes, this even concerns the chromatography that is used. Nevertheless, using several reconstitution mixtures might not be practical under most circumstances, since it requires enough of the typically scarce sample material for several sample preparations. It also makes it necessary to invest a lot more time, effort, and material in the workup procedure. It therefore appears to be more appropriate to use reconstitution solvents that is optimized for every chromatographical system that is used and agnostic to the analyzed chemical classes. As mentioned above, the choice of the reconstitution solvent can be challenging since UT are typically performed using reversed phase as well as normal phase columns with properties that are contrary to each other. The results of this study indicate that usage of methanol in the reconstitution solvent is beneficial for the analysis of blood plasma within the scope of this analysis workflow. Since the improvement of the peak areas of the investigated internal standard and the total feature count abated after using a methanol fraction of 30% we believe that this amount is most appropriate for further UT studies using the applied analysis workflow. Due to the fact that methanol has the second highest relative solvent strength in HILIC, higher amount of methanol might lead to unfavorable effects such as double peaks, especially at the beginning of the chromatography. The results of this study are clearly limited to the biomatrix and analytes investigated. It is therefore not the aim of this study to describe a reconstitution solvent mixture that is universally applicable. This kind of study needs to be performed for each workflow to evaluate its most appropriate reconstitution solvent.

## Conclusions

This study investigated the impact of different reconstitution mixtures on the peak area of tryptophan-d_5_, glucose, creatinine, palmitic acid, and the phophatidylcholine PC(P-16:0/P-16:0), as well as the total feature count of the analyses. While acetonitrile appears to be the first choice for the here described chromatographical conditions, it was shown that the used internal standard was almost not detectable at all. In most cases it was only detectable after adding methanol to the reconstitution solvent with increasing peak areas for higher amounts of methanol. Total feature count did also increase with an increasing amount of methanol used, leading to the conclusion that this effect is not only relevant for the investigated analytes, but several other analytes as well. Since the improvement of both parameters fades at 30% methanol, we recommend this amount for the here described workflow. These results are in line with another published study describing pure water as the most appropriate reconstitution solvent for human serum samples their chromatographical conditions merely using a reversed phase column.

Since systematic testing of the reconstitution solvent is not required in any of the guidelines describing quality insurance for UT, we recommend to incorporate this parameter. We also recommend systematically testing for the most appropriate reconstitution solvent for the investigated biomatrix and used internal standards in prior to conducting any studies.

## Electronic supplementary material

Below is the link to the electronic supplementary material.

Supplementary file1 (PDF 388 kb)
