# Supplementary material for: Impact of the used solvent on the reconstitution efficiency of evaporated biosamples for untargeted metabolomics studies
Source: Metabolomics. 2020 Mar 2;16(3):34. doi: 10.1007/s11306-019-1631-1 (PMC7052028; doi:10.1007/s11306-019-1631-1)
Supplement: Supplementary file 1 — Supplementary file1 (PDF 388 kb) [file 11306_2019_1631_MOESM1_ESM.pdf]

Impact of the used solvent on the reconstitution efficiency of evaporated biosamples for untargeted metabolomics studies

Electronic Supplementary Material

Sascha K. Manier, Markus R. Meyer

LC-HRMS/MS apparatus

The analysis was performed using a Thermo Fisher Scientific (TF, Dreieich, Germany) Dionex UltiMate 3000 RS pump consisting of a degasser, a quaternary pump, and an UltiMate autosampler, coupled to a TF Q-Exactive Plus system equipped with a heated electrospray ionization HESI-II source. Mass calibration was done prior to analysis according to the manufacturer's recommendations using external mass calibration. Additionally, before each experiment the apparatus' spray shield and capillary was cleaned. The performance of the column and the mass spectrometer was tested using a test mixture in prior to every experiment. Gradient reversed phase elution was performed on a TF Accucore PhenylHexyl column (100 mm × 2.1 mm, 2.6 µm) or a normal phase Macherey-Nagel (Düren, Germany) HILIC Nucleodur column (125 × 3 mm, 3 µm). The mobile phases for gradient elution using the PhenylHexyl column consisted of 2 mM aqueous ammonium formate containing acetonitrile (1%, v/v) and formic acid (0.1%, v/v, pH 3, eluent A), as well as 2mM ammonium formate solution with acetonitrile:methanol (1:1, v/v) containing water (1%, v/v) and formic acid (0.1%, v/v, eluent B). The flow rate was set from 0-10 min to 500 µL/min and from 10-13.5 min to 800 µL/min using the following gradient: 0-1.0 min 99% A, 1-10 min to 1% A, 10-11.5 min hold 1% A, 11.5-13.5 min hold 99% A. The gradient elution using the HILIC column was performed using aqueous ammonium acetate (200 mM, eluent C) and acetonitrile containing formic acid (0.1% , v/v, eluent D). The flow rate was set to 500 µL/min using the following gradient: 0-1 min 2% C, 1-5 min 20% C, 5-8.5 min 60% C, 8.5-10 min hold 60% C. 10-12 min hold 2% C. For preparation and cleaning of the injection system isopropanol:water (90:10, v/v) was used. The following settings were used: wash volume, 100 µL; wash speed, 4000 nL/s; loop wash factor, 2. Every analysis was performed at 40°C column temperature, maintained by a Dionex UltiMate 3000 RS analytical column heater. The injection volume for every analysis was 1 µL. The HESI-II source conditions for every experiment were as follows: ionization mode, positive or negative; sheath gas, 60 AU; auxiliary gas, 10 AU; sweep gas, 3 AU; spray voltage, 3.50 kV in positive mode and -4.0 kV in negative mode; heater temperature, 320°C; ion transfer capillary temperature, 320°C; and S-lens RF level, 50.0. Mass spectrometry was performed according to a previously optimized workflow using full scan (FS) only. The settings for FS data acquisition were as follows: resolution, 140,000 fwhm; microscans, 1; automatic gain control (AGC) target, 5×10^5; maximum injection time, 200 ms; scan range, *m/z* 50-750; polarity, negative or positive; spectrum data type, centroid. TF Xcalibur software version 3.0.63 was used for data handling. The analysis was performed using a randomized sequence order with five injections of pure methanol (PhenylHexyl column) or eluent D (HILIC column) samples at the beginning of the sequence for apparatus equilibration.

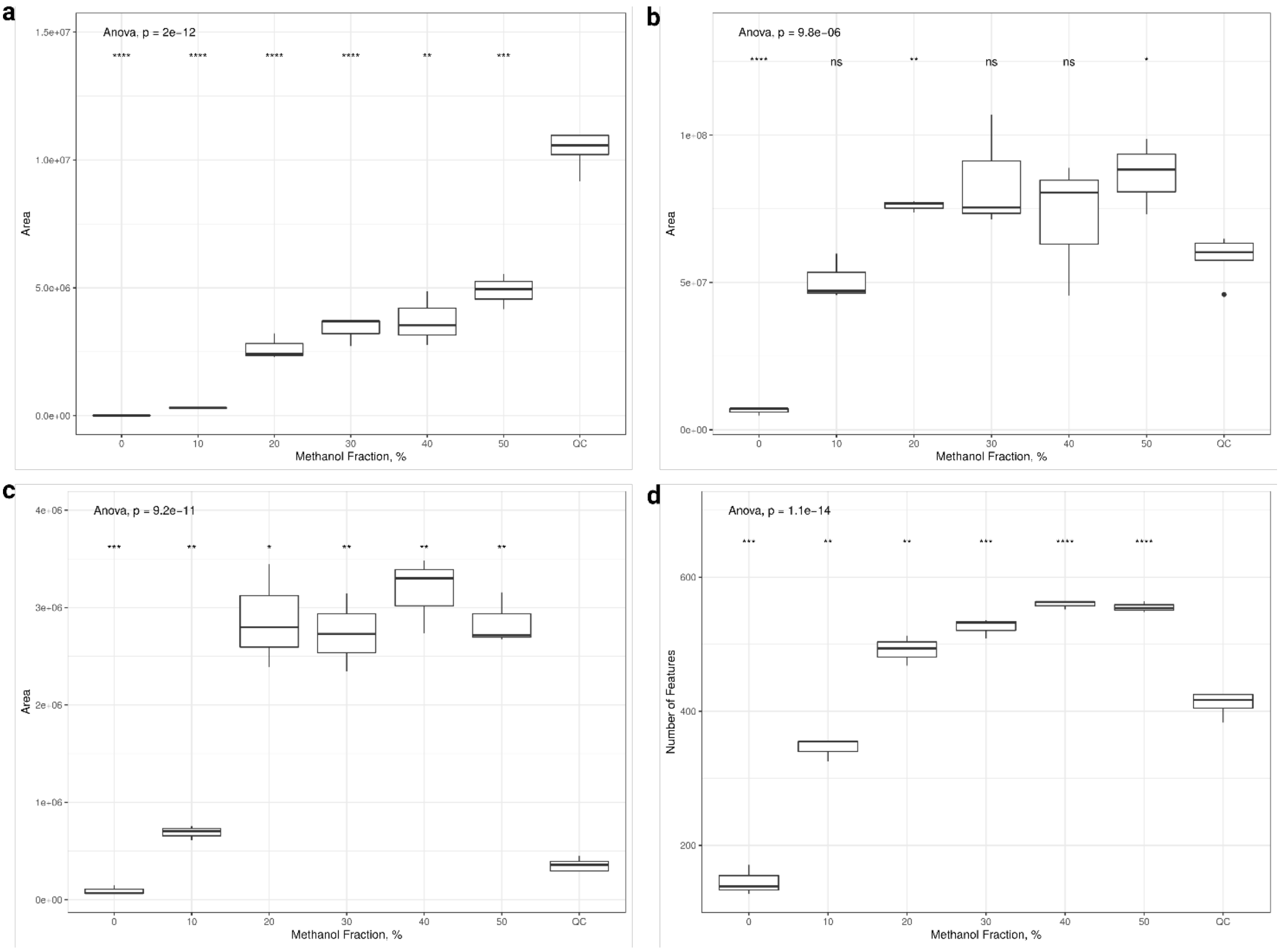

Fig. S1 Results of analysis using PhenylHexyl column in positive mode. Statistical evaluation was performed using one-way ANOVA and Welch's two sample t-test comparing each group to QC. **(a)** Tryptophan-d$_5$. **(b)** Glucose. **(c)**Palmitic acid. **(d)** Total feature count. ns = not significant; * = p < 0.05; ** = p < 0.01; *** = p < 0.001; **** = p < 0.0001

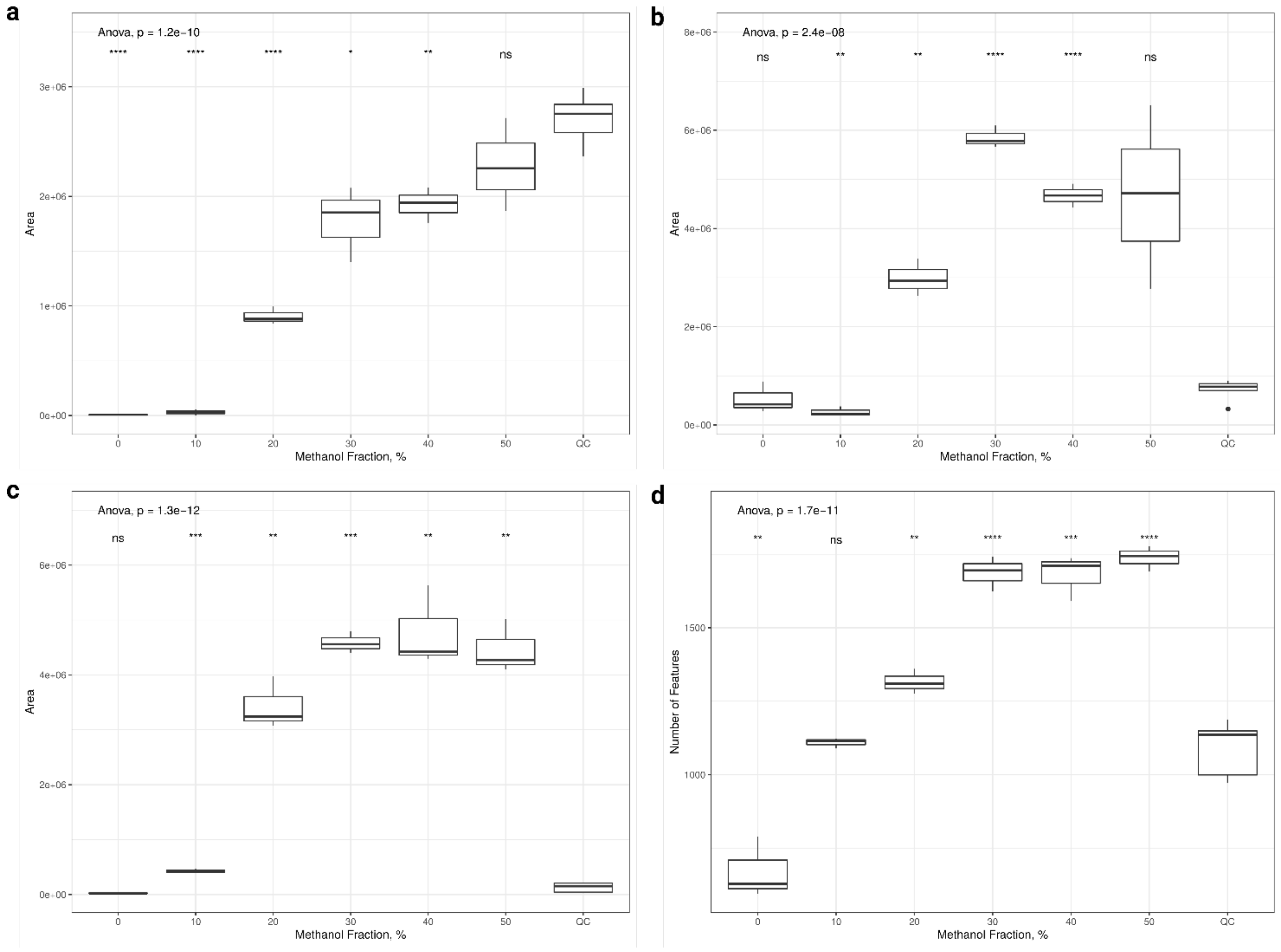

Fig. S2 Results of analysis using HILIC in positive mode. Statistical evaluation was performed using one-way ANOVA and Welch's two sample t-test comparing each group to QC. **(a)** Tryptophan-d_5. **(b)** Glucose. **(c)** Palmitic acid. **(d)** Total feature count. ns = not significant; * = $p < 0.05$; ** = $p < 0.01$; *** = $p < 0.001$; **** = $p < 0.0001$

Table S1 Peak picking and alignment parameters used for preprocessing. PH = PhenylHexyl, H = HILIC, pos = positive, neg = negative, ppm = allowed ppm deviation of mass traces for peak picking, snthresh = signal to noise threshold, mzdiff = minimum difference in m/z for two peaks to be considered as separate, prefilter 1 = minimum of scan points, prefilter 2 = minimum abundance, bw = bandwidth for grouping of peaks across separate chromatograms.

Column	Polarity	peakwidth, min	peakwidth, max	ppm	snthresh	mzdiff	prefilter 1	prefilter 2	bw
PH	pos	8.9	42	1.5	35	-0.008	7	10000	0.2
PH	neg	7.8	85	1.0	41	0.012	7	10000	1.0
H	pos	7.8	85	1.0	9	0.026	8	6200	1.0
H	neg	8.5	77	1.5	8	0.004	6	10000	1.5
